# Pan-cancer analysis of the prognostic and immunological role of SNX29: a potential target for survival and immunotherapy

**DOI:** 10.1186/s12920-023-01466-2

**Published:** 2023-02-24

**Authors:** Chengfei Xu, Fanghan Li, Zilin Liu, Chuanjing Yan, Jiangwei Xiao

**Affiliations:** 1 Department of Gastrointestinal Surgery, Clinical Medical College and The First Affiliated Hospital of Chengdu Medical College, Chengdu, 610500 People’s Republic of China; 2 School of Clinical Medicine, Chengdu Medical College, Chengdu, 610500 People’s Republic of China; 3 First Affiliated Hospital of Chengdu Medical College, Chengdu, 610500 People’s Republic of China

**Keywords:** Pan-cancer, Immune infiltration, Prognosis prediction, Microsatellite instability, Tumor mutation load, Drug sensitivity

## Abstract

**Background:**

There is growing evidence that the SNX family is critical for clinical prognosis, immune infiltration and drug sensitivity in many types of tumors. The relationships between the SNX29 gene and clinical prognosis as well as pan-cancer cell infiltration and drug sensitivity have not been fully elucidated.

**Methods:**

In the current study, we explored the correlation between SNX29 expression and 33 types of malignancies via TCGA and GTEx. The relationship between SNX29 expression and prognostic outcome in the pan-caner cohort was also analyzed. Immune infiltration, microsatellite instability, tumor mutational burden and potential therapeutic targets of SNX29 were investigated by analyzing public databases.

**Results:**

The expression of SNX29 was found to be significantly upregulated in most tumor tissues compared to normal tissues. SNX29 expression was associated with prognosis and clinical stage. In the immune infiltration analysis, a significant relationship was found between SNX29 expression and the level of immune infiltration. In addition, we found associations between the SNX29 gene and tumor mutation burden, microsatellite instability, immunoinhibition-related genes and autophagy-related genes. Finally, the expression of SNX29 was significantly associated with the sensitivity of various tumor cell lines to 8 antitumor drugs. These results suggest that SNX29 expression is important in determining the progression, immune infiltration and drug sensitivity of various cancers.

**Conclusion:**

This study provides novel insights into the potential pan-cancer targets of SNX29.

**Supplementary Information:**

The online version contains supplementary material available at 10.1186/s12920-023-01466-2.

## Introduction

Based on the World Health Organization (WHO) statistics for 2019, malignant neoplasms ranked the first or second leading cause of death before the age of 70 in 112 countries [1, 2]. Deaths caused by malignant tumors are increasing year by year [3]. Overall, the burden of cancer morbidity and mortality is growing dramatically worldwide and is imposing a heavy burden on people and national economies [4]. Tumorigenesis is multifactorial and involves the activation or suppression of oncogenes, immune escape, chromosomal instability, epigenetic alterations and abnormal cell signaling and [5]. To date, comprehensive treatment, including surgery, chemotherapy, radiotherapy and targeted therapy, is used for malignant tumors, but there is no way to achieve a complete cure [6]. Immunotherapy has recently emerged as a very promising treatment, especially immune checkpoint inhibitor treatment (e.g., PD1/PD-L1) [7]. Immune checkpoints play a critical role in the treatment of various tumors, including melanoma [8], colorectal cancer [9], gastric cancer [10] and lung cancer [11]. The Cancer Genome Atlas (TCGA) launched the Pan-Cancer Analysis Project on October 26–27, 2012, with the aim of obtaining commonalities, differences and emergent themes in the analysis of different cancer types and organs of origin [12]. We can use TCGA to evaluate and discover new immunotherapeutic targets made possible by the pan-cancer expression analysis of genes [13].

Sorting nexin 29 (SNX29) which maps to chr16p13.13-p13.12 is a protein-coding gene that is a member of the sorting nexin (SNX) family [14]. SNX proteins are widely distributed from yeast to mammals [5]. To date, 33 mammalian SNXs have been identified [15]. Sorting nexins contain the SNX-PX domain and play an important role in the endocytosis of cargo and endosomal trafficking pathways [16]. The PX domain, consisting of the phagocytic oxidase (phox) homology (PX) domain of amino acids 100–130, binds to phosphatidylinositol 3-phosphate (PI3P) [17]. Aberrant SNX proteins have been found to be associated with a variety of cancer diseases. Zhan et al. found that upregulation of SNX1 inhibited the growth, migration, and invasion and promoted the apoptosis of gastric cancer cells, in contrast to downregulation of SNX1, which dramatically enhanced cell growth, migration, and invasion and reduced apoptosis [18]. Sulin Zhang et al. demonstrated that SNX10 controls SRC levels by mediating autophagosome-lysosome fusion and SRC recruitment for autophagic degradation, thereby promoting the initiation and progression of colorectal cancer in mice [19]. Gu Jie Wu et al. showed that SNX20 expression was downregulated in most cancer types and was related to poor prognosis in lung adenocarcinoma by bioinformatics analysis [20]. These discoveries indicate that SNXs may be strategic potential therapeutic tumor targets for future development. SNX29 has not been reported in tumor-related studies. In recent years, there has been a small amount of research on SNX29 in human psychiatric diseases and the cardiovascular system. SNX29 is associated with mental disorders in the Han population, where SNP analysis of SNX29 showed that rs3743592 was significantly associated with major depressive disorder, while bipolar disorder (BPD) was significantly associated with BPD [21]. SNX29 may serve as a novel biomarker for the diagnosis of vasoresponsive pulmonary arterial hypertension [22]. However, there is still very little research on SNX29 in tumors. Thus, there is a need to investigate SNX29 across cancers.

In this study, we performed a pan-cancer analysis of SNX29 using TCGA, Genotype-Tissue Expression (GTEx), Cancer Cell Line Encyclopedia (CCLE) and CellMiner databases. Among the 33 cancers investigated, SNX29 gene expression was significantly different between normal and tumor samples in 14 cancers. We explored five cancers in which the SNX29 gene was strongly associated with patient survival. Subsequently, we investigated the associations between the SNX29 gene and immune characteristics, tumor mutational burden (TMB) and microsatellite instability (MSI). We also explored the relationship between the SNX29 gene and drug sensitivity. Our study initially revealed the potential application of the SNX29 gene in tumors.

## Materials and methods

### Data acquisition

The TCGA database (https://portal.gdc.cancer.gov/), a keystone of the cancer genomics project, is currently the largest database of cancer gene information, and it contains gene expression data, copy number variant data, SNP data and other data [23]. In the present study, TCGA raw mRNA expression datasets were obtained from the TCGA database, and the corresponding clinical characteristics were downloaded from the University of California Santa Cruz (UCSC) cancer genome browser (https://tcga.xenahubs.net). The raw TCGA data were normalized by fragments per kilobase of exon model per million mapped fragments (FPKM). The expression data of TCGA and GTEx (https://commonfund.nih.gov/GTEx) were merged and batch corrected using the normalizeBetweenArrays function, so that the gene expression data of TCGA and GTEx were at the same expression level. Data from each tumor cell line were downloaded from the CCLE database (https://portals.broadinstitute.org/ccle/) from each tumor cell line.

### Analysis of SNX29 expression differences

The expression matrix from the TCGA database and clinical features from the UCSC database were merged using patient ID. The Human Protein Atlas (HPA) is a website (https://www.proteinatlas.org/) that integrates multiomics technologies to map human proteins in cells, tissues and organs [24]. Therefore, we used the HPA database to illustrate the distribution of SNX29 protein in normal and cancer tissues.

### Relationship between SNX29 expression and prognosis, clinical stage or diagnosis

PrognoScan (http://www.prognoscan.org/) is an online meta-analysis tool [25]. We used it to analyze three datasets including GSE14333, GSE9195 and GSE30929. GSE14333 contained 290 primary colorectal cancer samples [26]. GSE9195 with 77 breast cancers samples from Canada was included for overall survival (OS) prediction [27]. GSE14333 contained 140 human liposarcoma specimens [2]. The relationships of gene expression with the OS and progression-free survival (PFS) of patients were further investigated. Survival analysis (*p* < 0.05) for each cancer type was performed using the Kaplan–Meier method, with the “survival” and “survminer” in R. In addition, we explored the relationship between SNX29 expression and pan-cancer prognosis by performing Cox regression analysis, and the results were visualized using the “survival” and “forestplot” in R. Clinicopathological correlations were performed using the “limma” and “ggpubr” packages. To explore the association of SNX29, age, stage and gender linked with survival status, we performed multivariate Cox regression survival analysis with the “survival” package. We used receiver operating characteristic (ROC) curves to assess the diagnostic value of SNX29 expression in various tumors.

### SNX29 expression and tumor immunity

CIBERSORT is a deconvolution algorithm that estimates the 22 phenotypes of human immune cells. The ESTIMATE algorithm generates three scores based on single-sample gene set enrichment analysis (ssGSEA) and is a novel tool for predicting stromal scores, immune scores and ESTIMATE scores. We explored 22 phenotypes of human immune cells in 33 cancers by CIBERSORT and further analyzed the relationshiop between SNX29 expression and the 22 immune cell phenotypes. In addition, we generated 3 scores, including the stromal score, immune score and ESTIMATE score, in each of the 33 cancers using ESTIMATE. We further analyzed the correlation between SNX29 expression and these 3 scores.

### Relationship between SNX29 expression and TMB or MSI

TMB and MSI are associated with sensitivity to immune checkpoint inhibitors. In this study, TMB was defined by calculating the frequency of variants and the number of variants/exon length for each tumor sample and dividing the total length of the protein-coding region by the nonsynonymous mutation sites. Values of MSI for each TCGA patient were derived from previously published studies [28]. Correlation analysis between cancer gene expression and TMB or MSI was performed by Spearman’s method. The “cor.test” command was used. Both metrics were displayed by radar plots.

### Relationship between SNX29 expression and autophagy-related genes and immunoinhibition-related genes

Coexpression analyses of SNX29 with immunoinhibition-related genes and autophagy-related genes were performed with the R package “limma”. Visualization was performed with the R packages “reshape2” and “RColorBrewer”.

### Tumor immunotherapy response and SNX29 expression

Tumor immune dysfunction and exclusion (TIDE) can be used to forecast the response to immune checkpoint blockade (ICB) through expression profiling [29]. Assessment of the response to ICB therapy in our current study were primarily conducted through TIDE (http://tide.dfci.harvard.edu/).

### GSEA enrichment analysis

We used the GSEA to assess the possible underlying mechanisms of SNX29. The number of random sample permutations was 100, the significance cutoff value was *p* < 0.05, and the enrichment map was plotted using Bioconductor and R software to visualize our results.

### SNX29 and drug sensitivity analysis

The CellMiner database is based on 60 cancer cell lines listed through the National Cancer Institute's Center for Cancer Research (NCI), and the NCI-60 cell line is the most widely used cancer cell sample population for anticancer drug testing [30]. In this study, we downloaded NCI-60 drug sensitivity data as well as RNA-seq gene expression data and explored the relationship between genes and antitumor drug sensitivity by Pearson correlation analysis. Differences with *p* < 0.05 were considered significant.

### Statistical analysis

SNX29 expression levels in tumor and normal tissues were estimated by the *Wilcoxon rank-sum test*. For survival analysis, the disease-free survival time, hazard ratio (HR) and* p* values were calculated using univariate Cox regression analysis and multivariate Cox regression analysis. Kaplan–Meier analysis was used to study the survival time and disease-free survival time in patients stratified to high or low SNX29 expression level group based on the median expression level as the cutoff value.* P* < 0.05 was set as the threshold of significance for all statistical analyses. The SNX29 gene expression and clinical staging were compared using the *Kruskal–Walis test*. The SNX29 gene expression and immune infiltration, TMB, MSI and autophagy-related genes and immune checkpoint-related genes were estimated by the *Pearson correlation*. ICB treatment response and SNX29 expression were tested using *Wilcoxon rank-sum* test. SNX29 expression and various drugs tested by the *Pearson correlation*. *p < 0.05, **p < 0.01, ***p < 0.001 and ns > 0.05.

## Results

### SNX29 protein expression in HPA

**Fig. 1:**
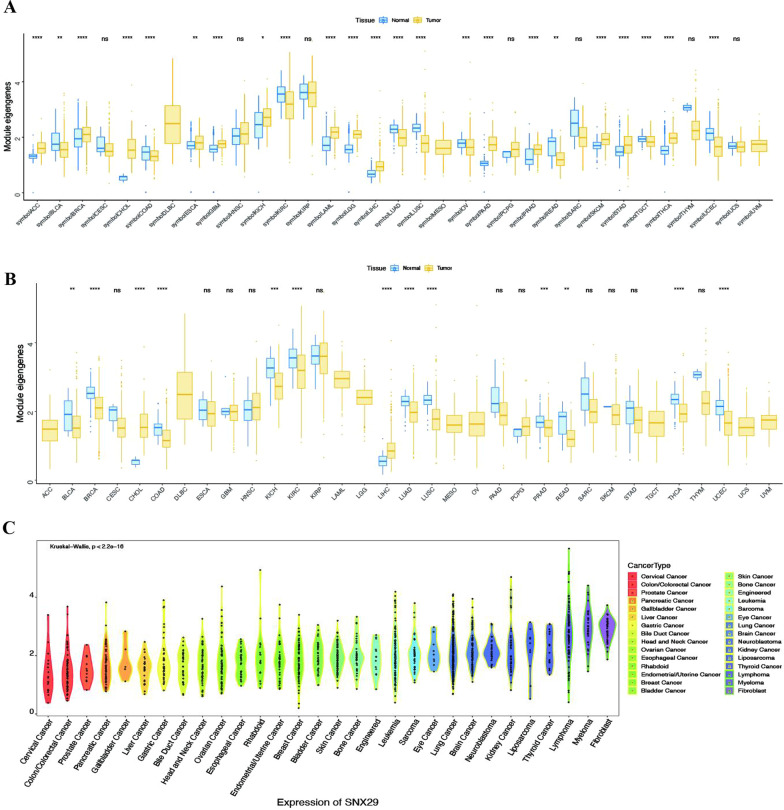
SNX29 mRNA expression in TCGA, GTEx and CCLE. **A** SNX29 mRNA expression levels in TCGA and GTEx databases for human pan-cancer analysis. **B** SNX29 mRNA expression levels in TCGA. **C**The mRNA expression of SNX29 in CCLE. *p < 0.05, **p < 0.01, ***p < 0.001 and ns > 0.05

To assess the SNX29 protein expression profile across cancers, we assessed SNX29 expression in various tumor and normal tissues using HPA. The outcomes demonstrated that SNX29 protein was widely expressed, with high expression in the testis, moderate expression in 25 tissues, such as the cerebral cortex, low expression in 15 tissues, such as the cerebellum, and no expression in 4 tissues, such as the prostate (Fig. 1A). Information on the expression of SNX29 protein in various cancers is summarized in Table 1.

**Table 1:** SNX29 protein expression in various cancers

Tissue	Number of samples	High expression	Medium expression	Low expression	Not detected
Glioma	12	1	3	8	0
lymphoma	12	0	4	2	6
Prostate cancer	11	0	3	5	3
Thyroid cancer	4	0	1	3	0
Head and neck cancer	4	0	1	2	1
Breast cancer	12	0	2	6	3
Renal cancer	12	0	2	5	5
Endometrial cancer	12	0	2	7	3
Liver cancer	10	0	1	7	2
Ovarian cancer	10	0	1	6	3
Colorectal cancer	11	0	1	10	0
Urothelial cancer	11	0	1	5	5
Testis cancer	11	0	1	2	5
Melanoma	12	0	1	6	
Lung cancer	10	0	0	8	2
Stomach cancer	9	0	0	3	6
Carcinoid	4	0	0	4	0
Pancreatic cancer	11	0	0	6	5
Cervical cancer	9	0	0	3	6
Skin cancer	11	0	0	7	4

### SNX29 gene expression across cancers

**Fig. 2:**
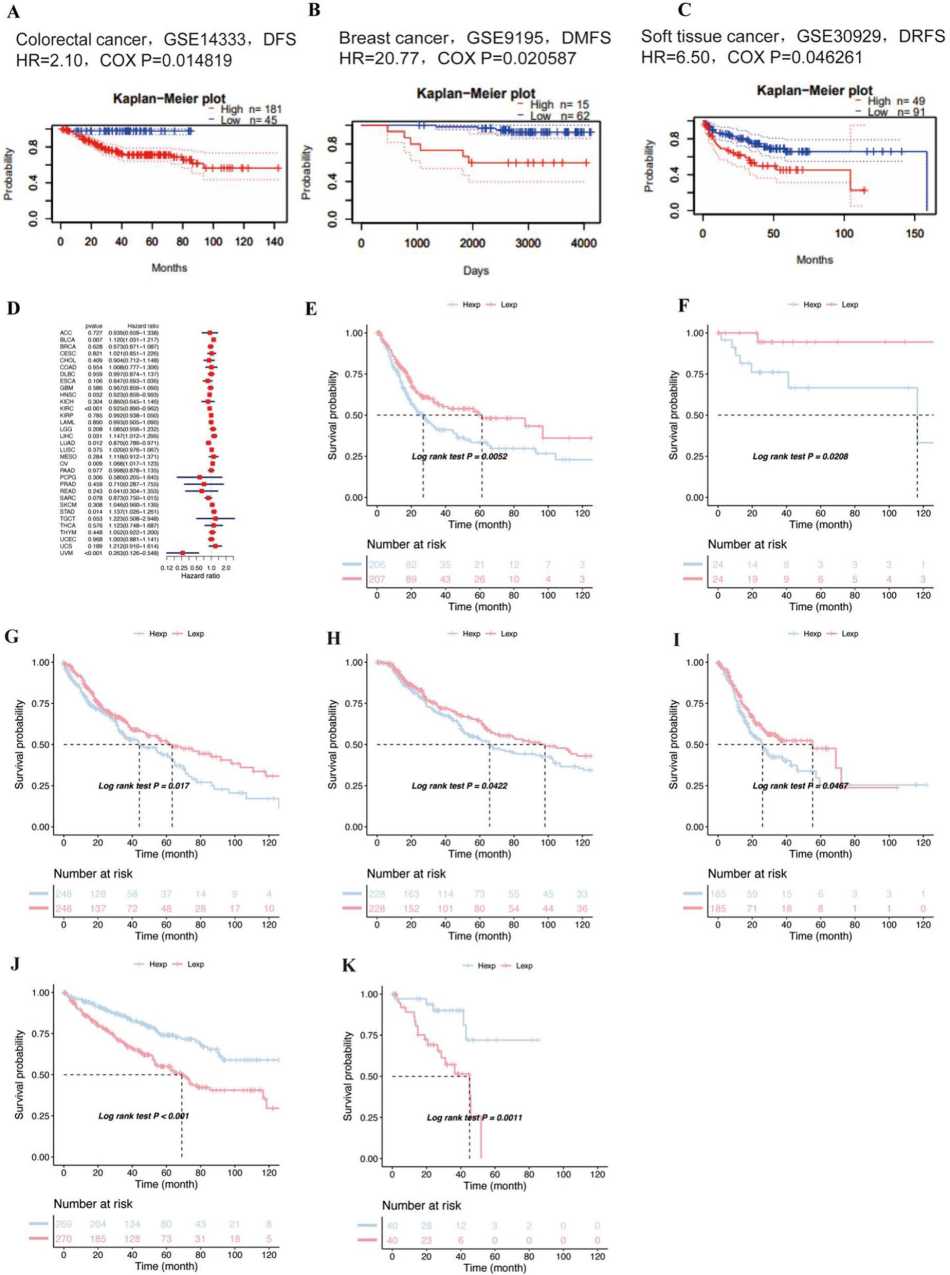
The relationship between SNX29 expression and DFS, DMFS, DRFS or OS. **A**–**C** The relationship between SNX29 expression and DFS, DMFS or DRFS in different cancers. **D** Forest plot illustrating the correlation between SNX29 expression and OS. **E**–**K** The relationship between SNX29 expression and OS was determined with Kaplan–19 Meier analysis. **E** BLCA, **F** DLBC, **G** LUSC, **H** SKCM, **I** STAD, **J** KIRC, **K** UVM. disease-free survival (DFS), distant metastasis-free survival (DMFS), distant recurrence-free survival (DRFS)

**Fig. 3:**
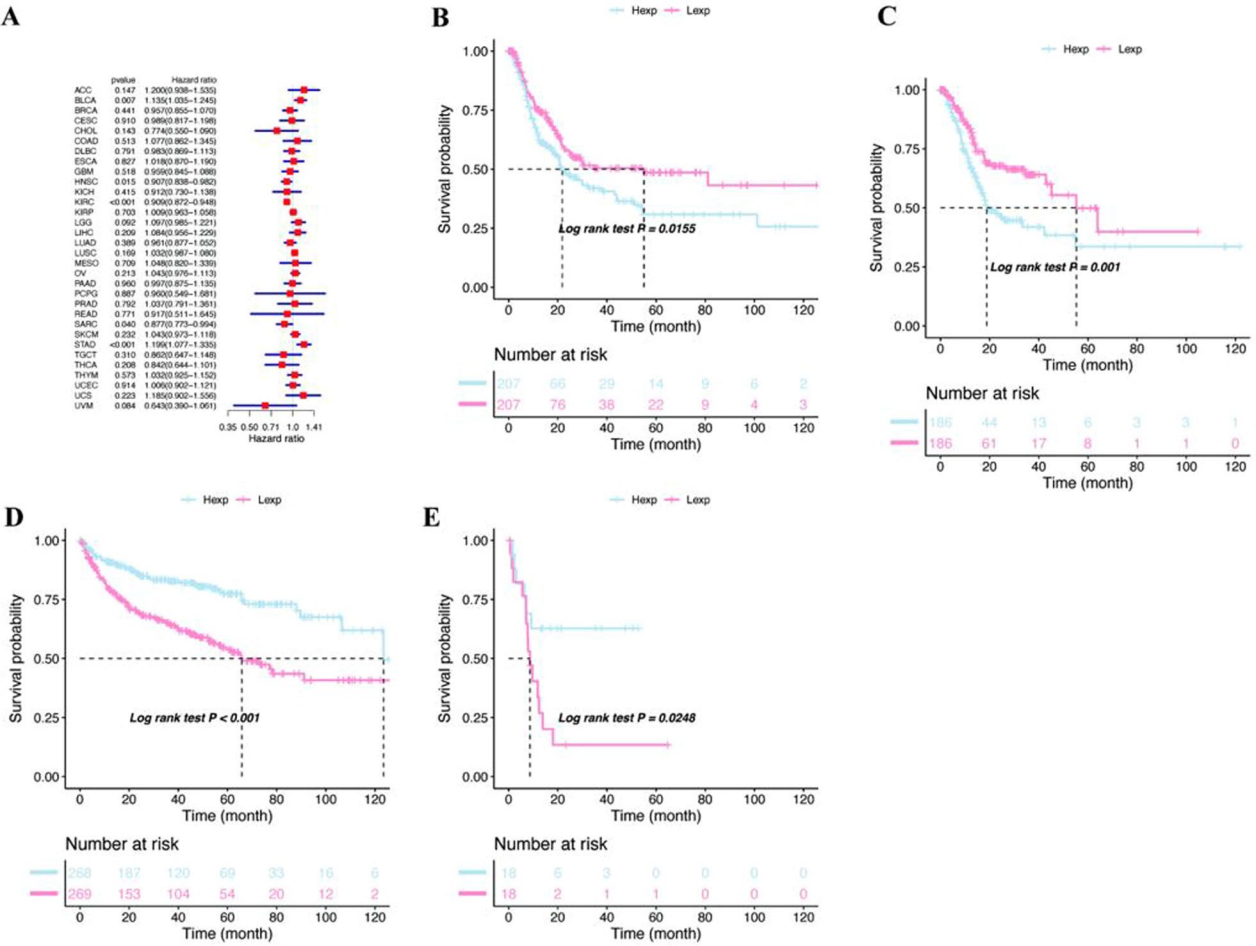
Relationship between SNX29 expression and PFS in patients. **A** Forest plot showing the relationship between SNX29 expression and PFS. **B**–**E** Comparison of the association between SNX29 expression and PFS using Kaplan–Meier analysis. **B** BLCA, **C** STAD, **D** KIRC, **E** CHOL

The expression of SNX29 in 33 human cancers was analyzed using the TCGA and GTEx datasets. The results showed that SNX29 expression was increased in 14 tumors, including adrenocortical carcinoma (ACC), breast invasive carcinoma (BRCA), cholangiocarcinoma (CHOL), esophageal carcinoma (ESCA), glioblastoma (GBM), kidney chromophobe (KICH), acute myeloid leukemia (LAML), brain lower grade glioma (LGG), liver hepatocellular carcinoma (LIHC), pancreatic adenocarcinoma (PAAD), prostate adenocarcinoma (PRAD), skin cutaneous melanoma (SKCM), stomach adenocarcinoma (STAD) and thyroid carcinoma (THCA), but decreased in bladder urothelial carcinoma (BLCA), colon adenocarcinoma (COAD), kidney renal clear cell carcinoma (KIRC), lung adenocarcinoma (LUAD), lung squamous cell carcinoma (LUSC), rectum adenocarcinoma (READ) and uterine corpus endometrial carcinoma (UCEC) (Fig. 1A). The expression of SNX29 in 33 human cancers was analyzed using the TCGA. The results showed that SNX29 expression was increased in 2 tumors, including CHOL and LIHC (Fig. 1B). The expression of SNX29 in different tumor cell lines in the CCLE expression profile is shown in Fig. 1C. Based on the survival analysis of the PrognoScan database, the survival curves of the 3 cohorts (GSE14333, GSE9195 and GSE30929) were significantly different (Fig. 2A–C). The prognosis of patients with high SNX29 expression is poor. In the OS analysis, Cox regression analysis revealed that high SNX29 expression was a protective factor for BLCA (*p* = 0.007), STAD (*p* = 0.014), ovarian serous cystadenocarcinoma (OV) (*p* = 0.009), and LIHC (*p* = 0.031). Conversely, it appeared to be a risk factor in head and neck squamous cell carcinoma (HNSC) (*p* = 0.032), KICH (*p* = 0.304), LUAD (*p* = 0.012) and uveal melanoma (UVM) (*p* = 0.001) (Fig. 2D). Kaplan–Meier analysis showed that patients with higher SNX29 levels had shorter OS in BLCA (*p* = 0.0052), lymphoid neoplasm diffuse large B-cell lymphoma (DLBC) (*p* = 0.0208), lung squamous cell carcinoma (LUSC) (*p* = 0.017), skin cutaneous melanoma (SKCM) (*p* = 0.0422), and STAD (*p* = 0.0467) (Fig. 2E–I). Patients with lower SNX29 expression showed longer OS in kidney renal clear cell carcinoma (KIRC) (*p* < 0.001) and UVM (*p* = 0.0011) (Fig. 2J–K). In progression-free survival (PFS) analysis, Cox regression demonstrated that high SNX29 expression was a protective factor in BLCA (*p* = 0.007) and STAD (*p* < 0.001). Meanwhile, it was a risk factor in HNSC (*p* = 0.015), KIRC (*p* < 0.001) and sarcoma (SARC) (*p* = 0.04) (Fig. 3A). The results of Kaplan–Meier survival analysis suggested that high expression of SNX29 was associated with poor PFS in BLCA (*p* = 0.0155) and STAD (*p* = 0.001) (Fig. 3B–C). Patients with high SNX29 expression levels showed longer OS in KIRC *(p* < 0.001) and CHOL (*p* = 0.0248) (Fig. 3D–E).

### Correlation analysis of SNX29 expression and pan-cancer clinical stage or diagnosis

**Fig. 4:**
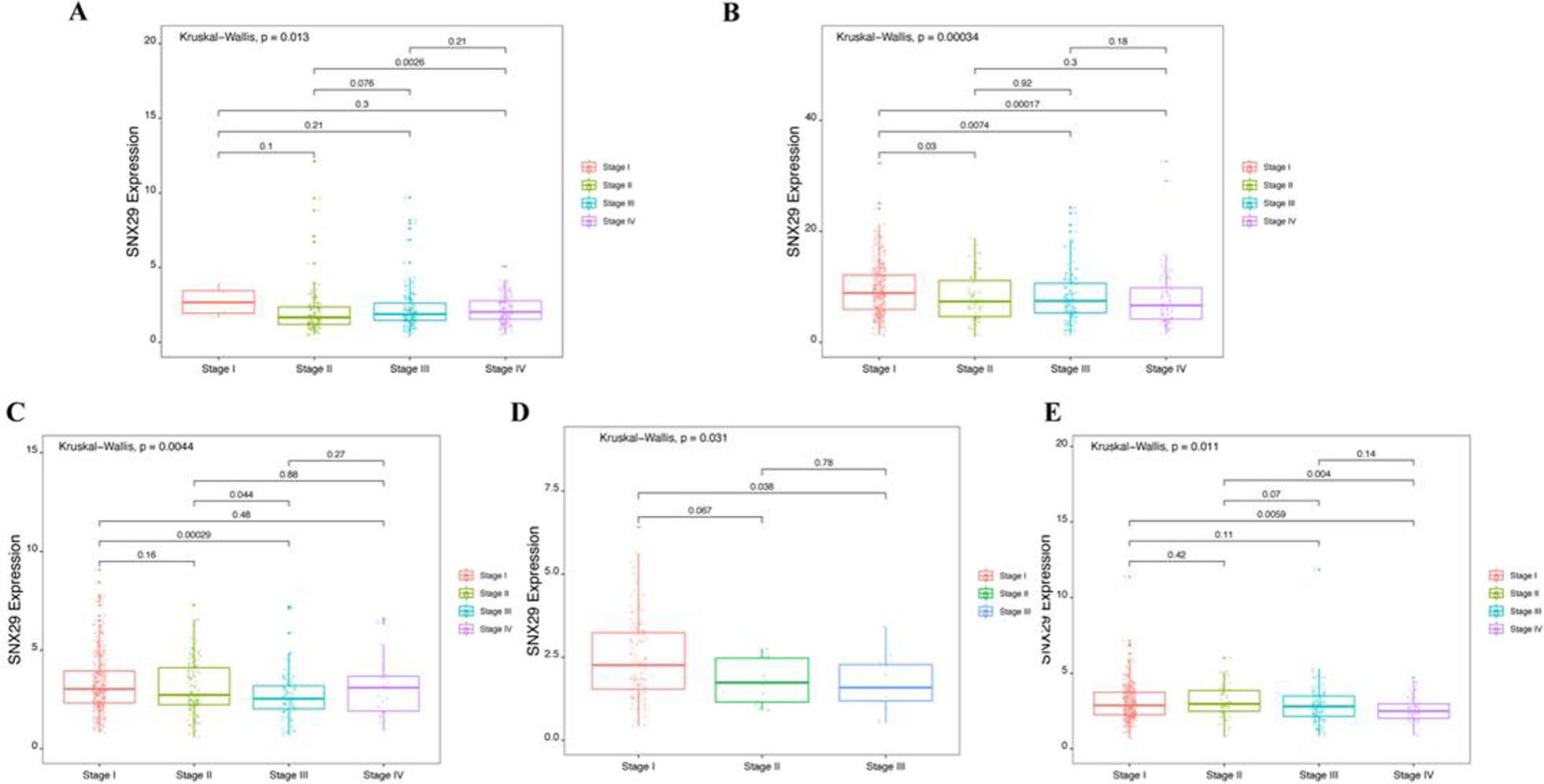
Relationship between SNX29 gene expression and clinical stage. **A** BLCA, **B** KIRC, **C** LUAD, **D** TGCT, **E** THCA

SNX29 expression was associated with the clinical stage of several cancers, including BLCA (*p* = 0.013), KIRC (*p* = 0.00034), LUAD (*p* = 0.0044), testicular germ cell tumor (TGCT) (*p* = 0.031), and THCA (*p* = 0.011) (Fig. 4). The expression of SNX29 was not significantly associated with clinical characteristics as shown in Additional file 1: Figure S1. The multivariate analysis results of stage, gender, age and SNX29 are presented in Additional file 1: Table S1. Stage was a risk factor for survival status in several cancers, including ACC, BLCA, BRAC, COAD, ECSA, HNSC, KICH, KIRC, KIRP, LGG, LIHC, LUAD and LUSC. Age was a risk factor for survival status in various cancers, including BLCA, COAD, GBM, KIRC and LGG. SNX29 was a risk factor for survival status in BLCA. We used ROC curves to assess the diagnostic value of SNX29 expression in various tumors. The area under the curve (AUC) values of SNX29 expression assessed by the ROC curves for various tumors were all less than 6 as shown in Additional file 1: Table S2.

### SNX29 expression and immune infiltration

**Fig. 5:**
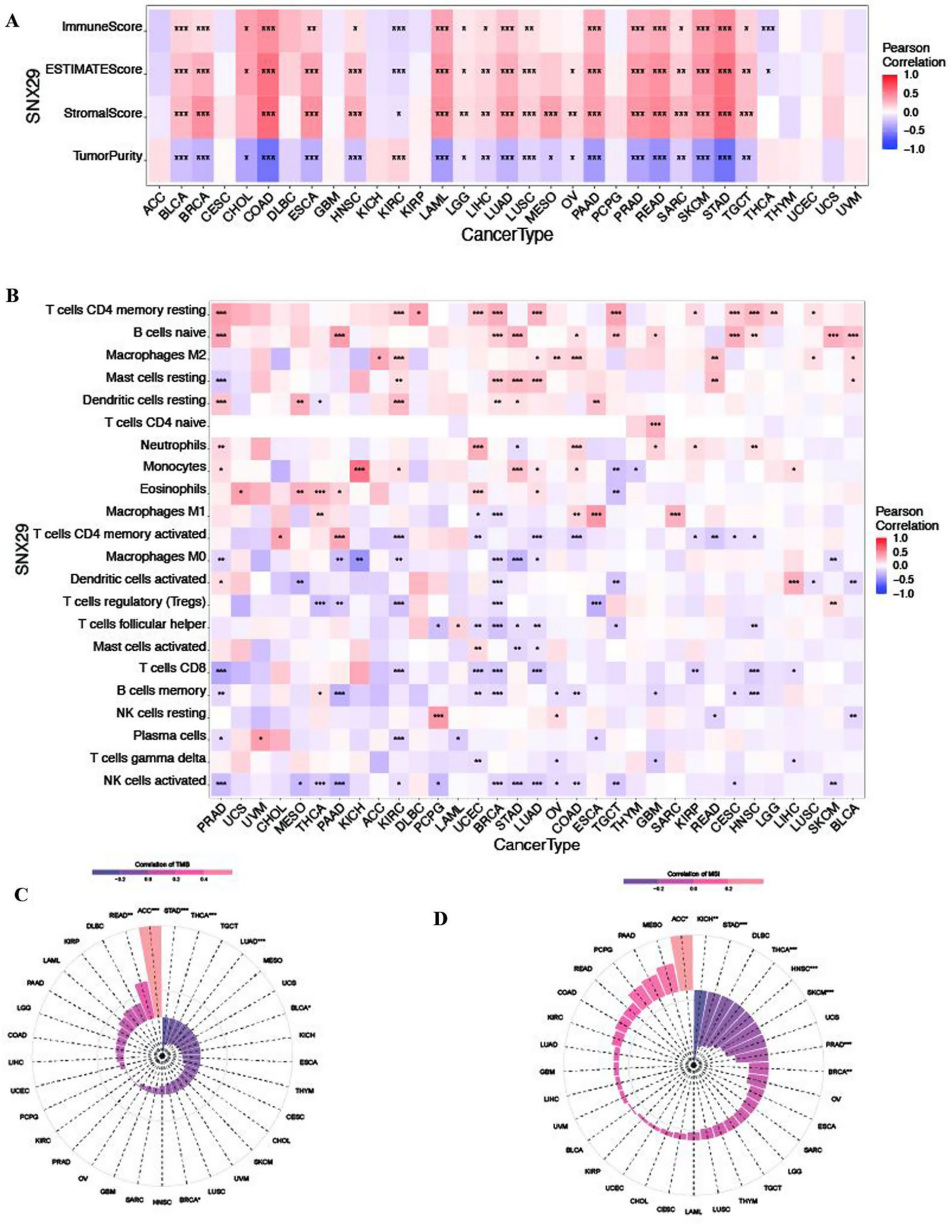
Relationship between SNX29 gene expression and immune infiltration, TMB and MSI. **A** Association between SNX29 expression and the stromal score, the immune score, the tumor purity and the ESTIMATE score. **B** Association between SNX29 expression and immune cell infiltration. **C** Radar plot demonstrating the correlation between TMB and SNX29 gene expression in various cancers. **D** Radar plot demonstrating the correlation between MSI and SNX29 gene expression.*p < 0.05, **p < 0.01, and ***P < 0.001

Our study showed significant positive correlations between SNX29 expression and stromal, immune and ESTIMATE scores in most cancers, such as BLCA, BRCA, COAD and LAML. In most tumors, SNX29 expression was associated with a decrease in tumor purity; this was the case for LAML, LUAD, READ and STAD (Fig. 5A). SXN29 expression was positively correlated with resting memory CD4 T cells in PRAD, KIRC, DLBC, UCEC, BRCA, LUAD, TGCT, kidney renal papillary cell carcinoma (KIRP), cervical squamous cell carcinoma and endocervical adenocarcinoma (CESC), HNSC, LGG and LUSC but negatively correlated with resting memory CD4 T cells in KIRP. Moreover, SXN29 expression was positively associated with B cells in PRAD, PRRD, BRCA, STAD, COAD, TGCT, GMB, CESC, HNSC, SKCM and BLCA. Similarly, SXN29 expression was positively associated with M2 macrophages in ACC, KIRC, LUAD, OV, COAD, ESCA, READ, LUSC and BLCA. Resting mast cells were positively correlated with SXN29 expression in KIRC, BRCA, STAD, OV, READ and BLCA but negatively associated with SXN29 expression in PRAD. Resting dendritic cells were positively correlated with SXN29 expression in PRAD, mesothelioma (MESO), THCA, KIRC, BRCA, STAD and ESCA but negatively correlated with SXN29 expression in THCA. Furthermore, naive CD4 T cells were positively associated with SXN29 expression in GBM. Neutrophils were positively correlated with SXN29 expression in PRAD, UCEC, COAD, GBM, KIRP and HNSC but negatively correlated with SXN29 expression in STAD. Monocytes were negatively correlated with SXN29 expression in TGCT and THYM but positively correlated with SXN29 expression in PRAD, KICH, STAD, LUAD, COAD and LIHC. SXN29 expression was positively correlated with eosinophils in UCS, MESO, THCA, PAAD, UCEC and LUAD but negatively correlated in TGCT. M1 macrophages were positively associated with SXN29 expression in THCA, COAD, ESCA and SARC but negatively associated with SXN29 expression in UCEC and BRCA. SNX29 expression was positively associated with CD4 memory T-cell activation in CHOL and PAAD but negatively related with CD4 memory T-cell activation in KIRC, LUAD, COAD, KIRP, READ, CESC and HNSC. SNX29 expression was negatively correlated with M0 macrophages in PRAD, PAAD, KICH, KIRC, BRCA, STAD, LUAD and SKCM. SNX29 expression was negatively correlated with dendritic cell activation in MESO, BRCA, TGCT, LUSC and BLCA but positively correlated with dendritic cell activation in PRAD and LIHC. SNX29 expression was negatively associated with regulatory T cells (Tregs) in THCA, PAAR, KIRC, BRCA and ESCA but positively associated with Tregs in SKCM. T-cell follicular helper cells were negatively associated with SNX29 expression in PCPG, UCEC, BRCA, STAD, LUAD, TGCT and HNSC but positively associated with SNX29 expression in LAML. SNX29 expression was negatively correlated with mast cell activation in STAD and LUAD but positively correlated with mast cell activation in UCEC. SNX29 expression was negatively correlated with CD8 T cells in PRAD, KIRC, UCEC, BRCA, LUAD, KIRP, HNSC and LIHC. SNX29 expression was negatively correlated with memory B cells in PRAD, PAAD, UCEC, BRCA, OV, COAD, GBM, CESC and HNSC but positively correlated with memory B cells in THCA. SNX29 expression was negatively correlated with resting NK cells in READ and BLCA but positively correlated with resting NK cells in PCPG and OV. SNX29 expression was negatively correlated with plasma cells in PRAD, KIRC, LAML and ESCA but positively correlated with plasma cells in UVM. Gamma delta T cells were negatively correlated with SNX29 expression in UCEC, OV, GBM and LIHC. Activated NK cells were negatively related to SNX29 expression in PRAD, MESO, THCA, PAAD, KIRC, BRCA, STAD, LUAD, OV, COAD, TGCT, CESC and SKCM (Fig. 5B).

### Associations of pan-cancer expression with TMB and MSI

In this study, we explored the relationships between SNX29 expression and TMB and MSI. For TMB, we found that SNX29 expression was positively correlated with TMB in READ and ACC. In contrast, SNX29 expression was negatively correlated with TMB in STAD, THCA and BRCA (Fig. 5C). For MSI, the results showed that SNX29 expression was positively correlated with MSI in ACC. However, SNX29 expression was negatively correlated with MSI in KICH, STAD, HNSC, SKCM, PRAD and BRCA (Fig. 5D).

### Pan-cancer expression of SNX29 and immunoinhibition-related genes and autophagy-related genes

**Fig. 6:**
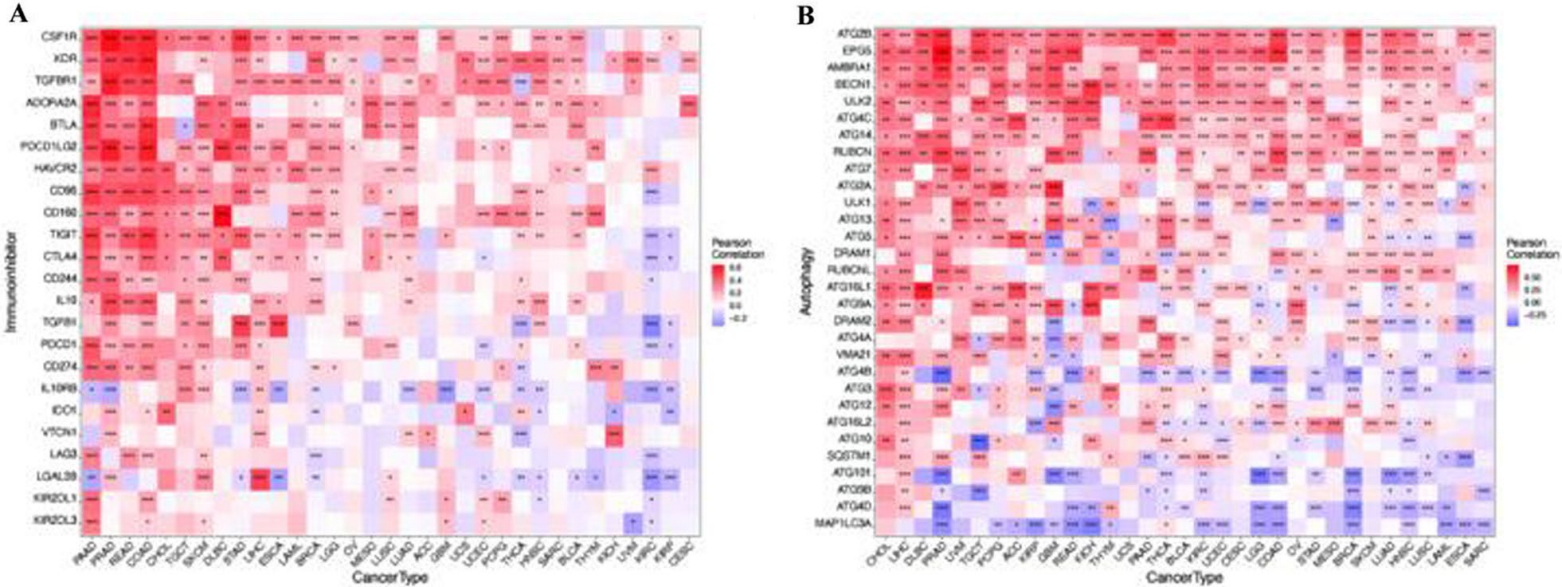
Heatmap of SNX29 expression in relation to autophagy-related genes and immune checkpoint-related genes across cancers. **A** SNX29 expression in relation to autophagy-related genes. **B** SNX29 expression in relation to immune checkpoint-related genes. *p < 0.05, **p < 0.01 and ***p < 0.001

Reviewing the relevant literature, we found that the SNX family plays important roles in autophagy and immunity [31–34]. Therefore, we speculated that SXN29 may have potential roles in immunity and autophagy. To explore the intrinsic mechanism of SNX29 expression in immunoinhibition-related genes, we analyzed the correlation between SNX29 expression and immunoinhibition-related genes in 33 cancer types. Comprehensive coexpression profiles of SNX29 showed that SNX29 expression was significantly positively correlated with many immune checkpoints, such as CTLA4, PDCD1 (PD-1), and CD274 (PD-L1) (Fig. 6A). Autophagy plays an important role in tumors, so we explored the role of SNX29 in autophagy. We showed that SNX29 expression was associated with most genes in most cancers, such as ATG2B, EPG, AMBRA1, and BECN1, in most cancers (Fig. 6B).

### Tumor immunotherapy response and SNX29 expression

**Fig. 7:**
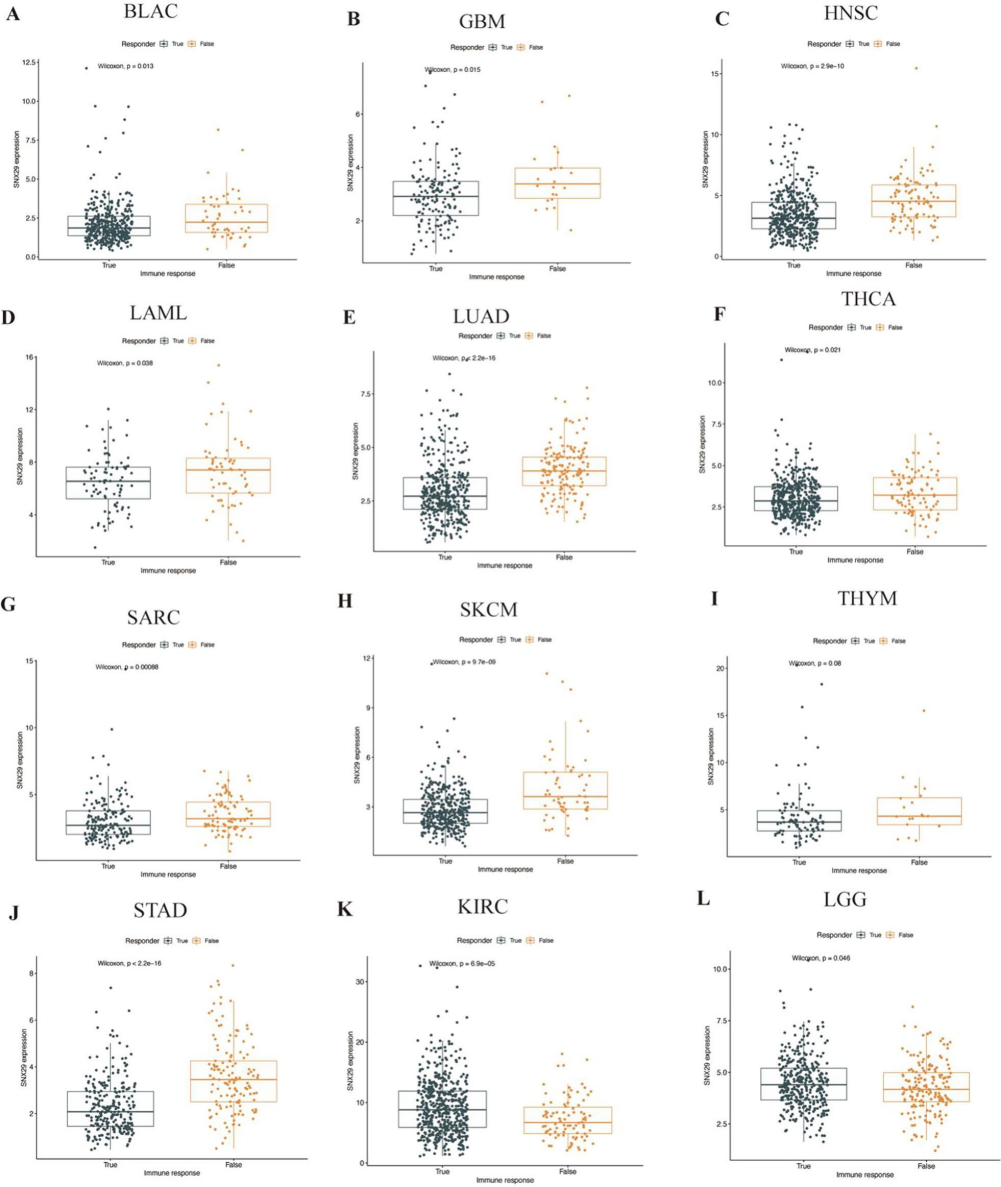
Relationship between ICB treatment response and SNX29 expression by TIDE. Relationship between ICB treatment response in TIDE and SNX29 expression. Low expression of SNX29 was strongly associated with ICB response, including **A** BLCA, **B** GBM, **C** HNSC, **D** LAML, **E** LUAD, **F** THCA, **G** SARC, **H** SKCM, **H** THYM and **J** STAD. High expression of SNX29 was negatively correlated with ICB response, including **K** KIRC and **L** LGG

Our study revealed that low SNX29 expression was strongly associated with ICB response in various cancers, including BLCA, GBM, HNSC, LAML, LUAD, THCA, SARC, SKCM, THYM and STAD (Fig. 7A–J). High SNX29 expression was negatively correlated with ICB response in some cancers, including KIRC and LGG (Fig. 7K–L).

### GSEA enrichment analysis

The possible underlying mechanisms of SNX29 expression were explored by GSEA. In HCC, we found significant enrichment of SNX29 in the KEGG terms: KEGG_FOCAL_ADHESION, KEGG_LYSOME, KEGG_MAPK_SIGNALING_PATHWAY, KEGG_PATHWAYS_IN_CANCER and KEGG_REGULATION_OF_ACTIN_CYTOSKELETON. The pan-cancer KEGG lists of SNX29 are available in Additional file 1: Table S3.

### Pan-cancer expression and drug sensitivity

**Fig. 8:**
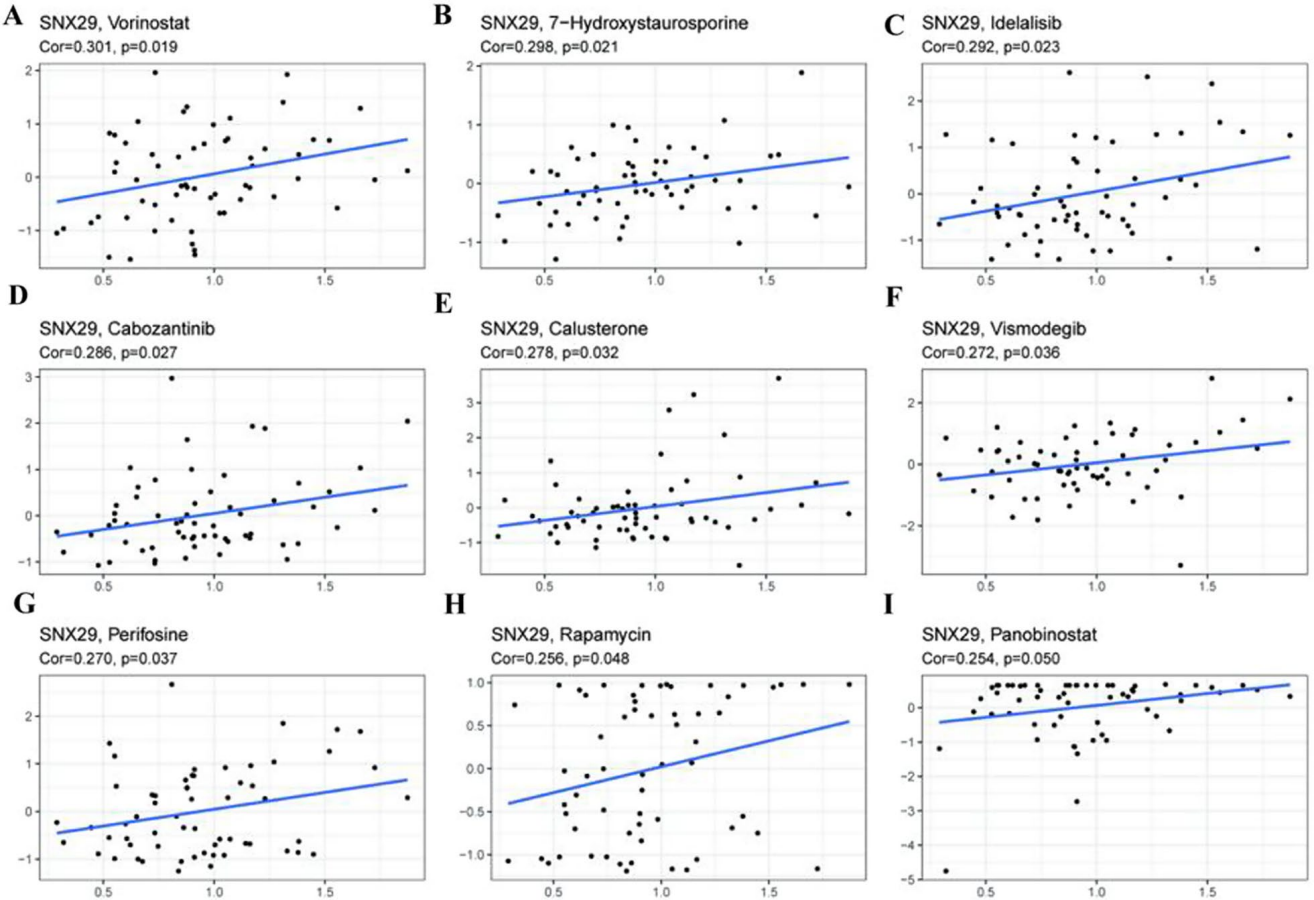
Correlation between SNX29 gene expression and drug sensitivity from NCI-60 cell line data. **A**–**I** Positive correlation scatter plots between SNX29 expression and various drugs tested by the Pearson correlation method using NCI-60 cell line data

We explored the correlation between the SNX29 gene and common antitumor drugs through the CellMiner database and found that high expression of the SNX29 gene was predicted to be associated with multiple antitumor drug tolerances. Various tumor cells with high SNX29 expression were found to be more sensitive to vorinostat, 7-hydroxystaurosporine, idelalisib, cabozantinib, calusterone, vismodegib, perifosine and rapamycin (Fig. 8).

## Discussion

Sorting nexin 29 (SNX29) is a member of the sorting factor family, which includes 33 mammalian SNXs [15]. Previous studies have shown that the SNX family plays an important role in a variety of cancers, such as pancreatic cancer [35], breast cancer [36], gastric cancer [37], colorectal cancer [38] and lymphoblastic leukemia [39]. There have been many pan-cancer studies in recent decades [40–44]. The relationship between SNX29 and multiple cancers has not been elucidated. Therefore, our current study integrated TCGA and GTEx analyses of SNX29 expression in 33 cancers in normal and tumor tissues. First, analysis based on TCGA and GTEx data showed that SNX29 was expressed at relatively higher levels in 14 different cancers compared to normal controls. Interestingly, SNX29 expression was decreased in tumor tissues in BRCA, COAD, LUSC, READ, SKCM and THCA. In addition, the OS analysis of Cox regression suggested that SNX29 expression was a protective factor in BLCA, STAD, OV, and LIHC but was a risk factor in HNSC, KICH, LUAD and UVM. Moreover, OS analysis showed that patients with increased SNX29 expression had shorter OS in BLCA, DLBC, LUSC, SKCM and STAD, while patients with decreased SNX29 expression showed longer OS in KIRC and UVM. In multivariate Cox regression, SNX29 expression is associated with prognosis in various tumors, including BLCA, KIRC, LUAD, OV, SARC and STAD. Beibei Hu et al. found that SNX29 expression was related to the prognosis of gastric cancer [45]. This result is consistent with our findings. SNX29 seemed to be the next research hotspot in various cancers. Based on the above evidence, we believe that SNX29 may be a potential new molecular biomarker for cancer diagnosis and prognosis that deserves further exploration.

To further elucidate the latent value of SNX29, we explored the association between SNX29 and immune cell infiltration by a bioinformatics approach. The SNX family exerts various effects on immune cell infiltration, because it plays a critical role in the host immune response. Eliran Ish-Shalom et al. demonstrated that decreased expression of SNX9 in colorectal cancer patients was inversely associated with bone marrow-derived suppressor cell levels [46]. Furthermore, Youde Yan et al. revealed that SNX10 deficiency increases the number of M2-type macrophages in experimental murine colitis [47]. Interestingly, our study showed that SNX29 expression was positively correlated with M2-type macrophage expression in a variety of tumors, such as ACC, KIRC, LUAD, OV, COAD, ESCA, READ, LUSC and BLCA, based on CIBERSORT analysis. Recently, many pan-cancer studies have used the ESTIMATE method as a metric to assess the prognosis of cancer patients in various tumors [48–50]. Zhe Gong et al. used tumor purity to predict the survival of gastric cancer and the relevance of immunotherapy [51]. In our current research, SNX29 expression showed a robust negative association with tumor purity based on ESTIMATE analysis in most cancers, such as LAML, LUAD, READ and STAD. In contrast, the immune score, ESTIMATE score and stromal score had significant positive correlations with SNX29 expression.

Cancer immunotherapy has made great clinical progress in the last decade in many types of tumors, such as melanoma [52], lung cancer [53] and hematologic tumors [54], especially in the field of ICB. ICB, such as PD-1/PD-L1 inhibitors, are promising treatments that can result in long-term remission in some cancer patients [54]. The SNX family plays a vital role in ICB therapies. Fan Lin-Wei et al. found that SNX20 expression was positively correlated with PD-L1 expression in lung cancer, and increasing the levels of SNX20 and PD-L1 resulted in a clinical response to PD-1 inhibitors in lung cancer [55]. Similarly, Chinmoy Ghosh et al. found that knockdown of SNX6 in cancer cells dramatically decreased PD-L1 protein levels, while deletion of SNX6 did not affect PD-L1 protein levels [56]. Our study showed that SNX29 expression was strongly positively correlated with PD-1 in 11 tumors. TMB levels and MSI-H demonstrate substantial neoantigen production and can serve as predictors of ICB response in many tumors. Dung T Le et al. showed that colorectal cancer with high microsatellite instability (MSI-H) was closely associated with high PD-1/PD-L1 expression and demonstrated a response to pembrolizumab (PD-1 blockade) [57]. In addition, F Wang et al. found that toripalimab (PD-1 blockade) exhibited antitumor activity in AGC patients, especially in combination with XELOX [58]. In the present study, we found that SNX29 expression was related to TMB and MSI in ACC, STAD and BRCA. The TIDE showed that low expression of SNX29 was strongly associated with ICB responses in various cancers, including BLCA, GBM, HNSC, LAML, LUAD, THCA, SARC, SKCM and THYM. High expression of SNX29 was closely associated with ICB responses, including KIRC and LGG. Thus, SNX29 could potentially serve as a predictor of the efficacy of immunotherapy in these cancer types. Taken together, the results of this study provide clues to the association between SNX29 and tumor immunotherapy.

Drug sensitivity has always been central to individualized cancer therapy. Drug sensitivity studies in cancer are essential to achieve personalized treatment of cancer patients and to advance precision medicine [59]. However, due to interindividual heterogeneity, large differences in drug sensitivity lead to the inefficient use of limited medical resources, so studies of molecules associated with drug response to optimize drug therapies are greatly needed. To date, there is no correlative study between SNX29 and medicine sensitivity or resistance. In our previous study, Pearson correlation analysis was performed between SNX29 mRNA expression and antitumor drug activity in NCI-60 cancer cell lines. The results showed that the SNX29 gene was significantly associated with the sensitivity of various tumor cell lines to nine antitumor drugs, including vorinostat, 7-hydroxystaurosporine, idelalisib, cabozantinib, calusterone, vismodegib, perifosine, rapamycin and panobinostat. Through KEGG analysis, our current study revealed that the focal adhesion pathway is the most common signaling pathway in which SNX29 is involved across cancers. Recent studies have shown that the focal adhesion pathway plays a critical role in drug resistance [31, 60, 61]. Takao Nakanishi et al. found that integrin and SFK in the focal adhesion pathway may be targets for promoting vinorelbine resistance [31]. Therefore, we speculate that the role of SNX29 in chemotherapy may be correlated with the focal adhesion pathway. Based on the above results, the detection of SNX29 gene expression has outstanding potential value for the selection of antitumor drugs.

The present study was the first to perform bioinformatics analysis of the SNX29 gene across cancers, and some shortcomings are worth considering. First, we performed bioinformatics analysis without in vitro or in vivo experiments to validate the predicted results. Second, multiple determinations of SNX29 expression and survival prognosis were made using TCGA data without validation with our own data. Further validation is needed through multicenter clinical studies is needed. Finally, although we have demonstrated a clear relationship between SNX29 gene expression and immune infiltration in multiple tumors, all using an indirect approach, single-cell sequencing may be needed to explore this further. There are still some limitations in this study. All tumor samples were used for analysis in TCGA. Tumor samples contain both primary and metastatic tumors, and primary and metastatic tumors have different biological characteristics that may lead to biased results.

## Conclusion

This pan-cancer study of SNX29 gene expression, prognostic assessment, immune infiltration and potential drug therapeutic targets in 33 tumors was conducted in a comprehensive and systematic manner. Our current study revealed that the expression of SNX29 was significantly increased in tumor tissues and was correlated with the prognosis and clinical staging across cancers. In addition, SNX29 gene expression was significantly correlated with the level of immune cell infiltration. Finally, the SNX29 gene was associated with the drug sensitivity of 9 antitumor agents. In summary, it is reasonable to conclude that SNX29 gene expression may be relevant to a variety of tumorigenesis factors, the tumor microenvironment, and drug sensitivity, providing new ideas for the development of new targets for the diagnosis, prognostic assessment, and treatment of various cancers.

## Supplementary Information

**Additional file 1. Figure S1.** Relationship between SNX29 gene expression and clinical stage in various tumors. **Table S1.** The multivariate analysis of stage, gender, age and SNX29. **Table S2.** AUC value in various tumors. **Table S3.** The pan-cancer KEGG lists of SNX29.

## Data Availability

The datasets analyzed during the current study are available in theTCGA (https://portal.gdc.cancer.gov/), GTEx (https://www.gtexportal.org/home/index.html), UCSC database(http://genome.ucsc.edu/),CellMiner database(https://discover.nci.nih.gov/cellminer/home.do), GEO (https://www.ncbi.nlm.nih.gov/geo/) and HPA database (https://www.proteinatlas.org/) databases.
