# Supplementary figures and images for: Pan-cancer analysis of the prognostic and immunological role of SNX29: a potential target for survival and immunotherapy
Source: BMC Med Genomics. 2023 Feb 24;16:34. doi: 10.1186/s12920-023-01466-2 (PMC9951530; doi:10.1186/s12920-023-01466-2)

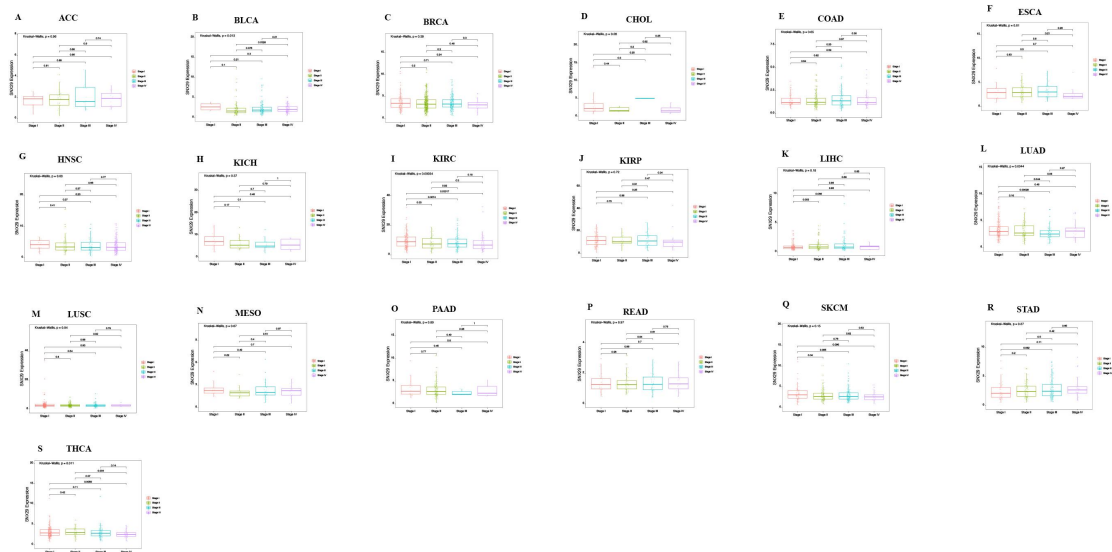

Figure S1 Relationship between SNX29 gene expression and clinical stage in various tumors.

Supplement: Supplementary file 1 — Additional file 1. Figure S1. Relationship between SNX29 gene expression and clinical stage in various tumors. Table S1. The multivariate analysis of stage, gender, age and SNX29. Table S2. AUC value in various tumors. Table S3. The pan-cancer KEGG lists of SNX29. [file 12920_2023_1466_MOESM1_ESM.zip › Supplementary material/Supplementary Figure S1.pdf]
